# Neuroendocrine carcinoma of uterine cervix findings shown by MRI for staging and survival analysis – Japan multicenter study

**DOI:** 10.18632/oncotarget.27613

**Published:** 2020-10-06

**Authors:** Kazuhiro Kitajima, Takako Kihara, Yusuke Kawanaka, Aki Kido, Kotaro Yoshida, Yasunari Mizumoto, Akiko Tomiyama, Shigeo Okuda, Masahiro Jinzaki, Fumi Kato, Junko Takahama, Akiko Takahata, Yoshihiko Fukukura, Atsushi Nakamoto, Tetsuya Tsujikawa, Jiro Munechika, Yoshimitstu Ohgiya, Nobuyuki Kawai, Satoshi Goshima, Ayumi Ohya, Yasunari Fujinaga, Takeru Fukunaga, Shinya Fujii, Masahiro Tanabe, Katsuyoshi Ito, Takahiro Tsuboyama, Yuichiro Kanie, Shigeaki Umeoka, Shintaro Ichikawa, Utaroh Motosugi, Sayaka Daido, Ayumu Kido, Tsutomu Tamada, Mitsuru Matsuki, Tsuneo Yamashiro, Koichiro Yamakado

**Affiliations:** ^1^Department of Radiology, Hyogo College of Medicine, Nishinomiya, Hyogo, Japan; ^2^Department of Surgical Pathology, Hyogo College of Medicine, Nishinomiya, Hyogo, Japan; ^3^Department of Diagnostic Radiology and Nuclear Medicine, Graduate School of Medicine, Kyoto University, Kyoto, Japan; ^4^Department of Radiology, Kanazawa University, Graduate School of Medicine Science, Kanazawa, Ichikawa, Japan; ^5^Department of Obstetrics and Gynecology, Kanazawa University, Graduate School of Medicine Science, Kanazawa, Ichikawa, Japan; ^6^Department of Radiology, Keio University School of Medicine, Tokyo, Japan; ^7^Department of Diagnostic and Interventional Radiology, Hokkaido University Hospital, Sapporo, Hokkaido, Japan; ^8^Department of Radiology, Nara Medical University, Nara, Japan; ^9^Department of Radiology, Kyoto Prefectural University of Medicine, Kyoto, Japan; ^10^Department of Radiology, Graduate School of Medical and Dental Sciences, Kagoshima University, Kagoshima, Japan; ^11^Department of Radiology, Osaka University Graduate School of Medicine, Suita, Osaka, Japan; ^12^Biomedical Imaging Research Center, University of Fukui, Fukui, Japan; ^13^Department of Radiology, Showa University School of Medicine, Tokyo, Japan; ^14^Department of Radiology, Gifu University Hospital, Gifu, Japan; ^15^Department of Diagnostic Radiology and Nuclear Medicine, Hamamatsu University School of Medicine, Hamamatsu, Shizuoka, Japan; ^16^Department of Radiology, Shinshu University School of Medicine, Matsumoto, Nagano, Japan; ^17^Division of Radiology, Department of Pathophysiological and Therapeutic Sciences, Tottori University, Tottori, Japan; ^18^Department of Radiology, Yamaguchi University Graduate School of Medicine, Yamaguchi, Japan; ^19^Department of Radiology, National Hospital Organization Osaka National Hospital, Osaka, Japan; ^20^Department of Radiology, Japanese Red Cross Society Himeji Hospital, Himeji, Hyogo, Japan; ^21^Department of Radiology, Japanese Red Cross Wakayama Medical Center, Wakayama, Japan; ^22^Department of Radiology, University of Yamanashi, Yamanashi, Japan; ^23^Department of Radiology, National Hospital Organization Kyoto Medical Center, Kyoto, Japan; ^24^Department of Radiology, Kawasaki Medical School, Okayama, Japan; ^25^Department of Diagnostic Radiology, Kindai University Faculty of Medicine, Osaka, Japan; ^26^Department of Radiology, Graduate School of Medical Science, University of the Ryukyus, Okinawa, Japan

**Keywords:** cervical cancer, neuroendocrine carcinoma, small cell carcinoma, large cell carcinoma, MRI

## Abstract

Objectives: To investigate neuroendocrine carcinoma (NEC) of the uterine cervix cases for MRI features and staging, as well as pathological correlations and survival.

Results: FIGO was I in 42, II in 14, III in 1, and IV in 5 patients. T2-weighted MRI showed homogeneous slightly high signal intensity and obvious restricted diffusion (ADC map, low intensity; DWI, high intensity) throughout the tumor in most cases, and mild enhancement in two-thirds. In 50 patients who underwent a radical hysterectomy and lymphadenectomy without neoadjuvant chemotherapy (NAC), intrapelvic T staging by MRI overall accuracy was 88.0% with reference to pathology staging, while patient-based sensitivity, specificity, and accuracy for metastatic pelvic lymph node detection was 38.5%, 100%, and 83.3%, respectively. During a mean follow-up period of 45.6 months (range 4.3–151.0 months), 28 patients (45.2%) experienced recurrence and 24 (38.7%) died. Three-year progression-free and overall survival rates for FIGO I, II, III, and IV were 64.3% and 80.9%, 50% and 64.3%, 0% and 0%, and 0% and 0%, respectively.

Materials and Methods: Sixty-two patients with histologically surgery-proven uterine cervical NEC were enrolled. Twelve received NAC. Clinical data, pathological findings, and pretreatment pelvic MRI findings were retrospectively reviewed. Thirty-two tumors were pure NEC and 30 mixed with other histotypes. The NECs were small cell type (41), large cell type (18), or a mixture of both (3).

Conclusions: Homogeneous lesion texture with obvious restricted diffusion throughout the tumor are features suggestive of cervical NEC. Our findings show that MRI is reliable for T staging of cervical NEC.

## INTRODUCTION

Neuroendocrine carcinomas (NECs) of the female genital tract are aggressive uncommon tumors that usually involve the uterine cervix and ovaries, though are very rarely seen in the endometrium [1]. A uterine cervix NEC is rare, accounting for only 1–6% of all cervical malignancies. According to the World Health Organization (WHO) classification, neuroendocrine tumors in the uterine cervix are categorized into 4 categories; typical carcinoid, atypical carcinoid, small cell neuroendocrine carcinoma (SCNEC), and large cell neuroendocrine carcinoma (LCNEC) [2]. The WHO defines SCNEC as an undifferentiated carcinoma with cellular and nuclear features that include small-sized cells, scant cytoplasm, hyperchromatic features, finely granular and molded nuclei, and inconspicuous nuclei. On the other hand, an LCNEC is defined as undifferentiated large cells that lack the cytologic and architectural features of small cell carcinoma, and show glandular or squamous differentiation. More simply, LCNEC is a malignant tumor composed of large cells that show neuroendocrine differentiation.

Cervical NEC has a higher frequency of lymphovascular invasion, lymphatic and distal metastasis, and recurrence as compared to other subtypes of uterine cervical malignancies, e. g., squamous cell carcinoma and adenocarcinoma [3, 4]. Clinical, Papanicolaou’s smear, and cervical biopsy findings are the primary screening and diagnostic tools for cervical malignancy cases. However, findings obtained in smear and cervical biopsy analyses are insensitive and inconclusive for diagnosis of cervical NEC in some patients, due to limited sample size or tumor heterogeneity [5]. Magnetic resonance imaging (MRI) is a well-established method for diagnosis and staging of uterine cervical tumors. Should MRI results be shown informative for correct diagnosis of NEC of the uterine cervix as well as accurate staging prior to performing treatment, the modality would be considered a useful clinical tool. To the best of our knowledge, 2 different reports have been presented that include discussion of MRI characteristics of uterine cervical SCNEC [6, 7], with a small number of patients in each (*n* = 7 and 26, respectively), while no known reports discussing MRI findings of cervical LCNEC are available. Therefore, features of uterine cervical NEC shown by MRI are not well recognized.

The aim of this study was to identify the distinct features and staging accuracy of uterine cervical NEC, including SCNEC and LCNEC, using MRI, as well as pathological correlations. Survival of affected patients was also evaluated.

## RESULTS

The clinicopathologic features of the present patients (*n* = 62) are summarized in Table 1. Their aged ranged from 26 to 82 years (median 39.5, mean 43.5 years), including 6 (9.7%) from 20–29 years old, 25 (40.3%) from 30–39, 15 (24.2%) from 40–49, 7 (11.3%) from 50–59, 5 (8.1%) from 60–69, 3 (4.8%) from 70–79, and 1 (1.6%) older than 79 years.

**Table 1:** Patient and tumor characteristics

Character	*N*	%
Age at diagnosis (years)		
Median (range)	39.5 (26–82)	
NEC		
pure type	32	51.6
SCNEC	21	33.9
LCNEC	8	12.9
Mixed SCNEC and LCNEC	3	4.8
mixed with other histotype	30	48.4
SCNEC with other histotype	20	32.2
LCNEC with other histotype	10	16.1
Positivity for synaptophysin	57/62	91.9
Positivity for chromogranin A	48/60	80.0
Positivity for CD56	42/53	79.2
FIGO stage		
IB1	32	51.6
IB2	10	16.1
IIA2	1	1.6
IIB	13	21.0
IIIA	1	1.6
IVB	5	8.1
Treatment		
Surgery	8	12.9
Surgery+chemotherapy	31	50.0
Surgery+chemotherapy+radiotherapy	11	17.7
NAC+Surgery	3	4.8
NAC+Surgery+chemotherapy	7	11.3
NAC+Surgery+chemotherapy+radiotherapy	2	3.2

*N*: number, NEC: neuroendocrine carcinoma, SCNEC: small cell neuroendocrine carcinoma, LCNEC: large cell neuroendocrine carcinoma, FIGO: Federation of Gynecology and Obstetrics, NAC: neoadjuvant chemotherapy.

Presenting symptoms were known for 62 patients and included vaginal bleeding (*n* = 44), abnormal discharge (*n* = 8), abnormal pap smear results (*n* = 6), and inadvertent discovery in an examination for another disease (*n* = 4). Abnormal serum tumor marker levels for NSE were seen in 13 (27.1%) of 48, proGRP in 3 (17.6%) of 17, CA125 in 6 (13.3%) of 45, CA19-9 in 5 (11.9%) of 42, CEA in 4 (7.1%) of 56, SCC in 4 (7.1%) of 56, AFP in 0 (0%) of 5, and CYFRA in 0 (0%) of 2 patients. Cervical biopsy results revealed neuron-specific enolase (NEC) in 47 (75.8%) of the total 62 patients.

Pathological findings indicated pure NEC in 32 (51.6%), including pure SCNEC in 21, LCNEC in 8, and a mixture of SCNEC and LCNEC in 3 patients. Of the remaining 30 patients (48.4%), SCNEC combined with other pathologies was seen in 20, such as squamous cell carcinoma, adenocarcinoma, serous carcinoma, adenosquamous cell carcinoma, or carcinoma *in situ*, while LCNEC combined with other pathologies was noted in 10, including squamous cell carcinoma, adenocarcinoma, endometrioid adenocarcinoma, mucinous endocervical adenocarcinoma, or carcinoma *in situ*.

All tumors were positive for at least 1 neuroendocrine marker (synaptophysin, chromogranin A, CD56). Immunohistochemical findings of the specimens showed positive reactions for synaptophysin in 57 (91.9%) of 62, chromogranin A in 48 (80.0%) of 60, CD56 in 42 (79.2%) of 53, NSE in 5 (83.3%) of 6, p53 in 6 (75.0%) of 8, and SSRT2a in 2 (100%) of 2 patients examined for neuroendocrine markers. In addition, Ki-67 labelling index was identified in 16, ranging from 30% to 90% with a median of 80%. Also, the mitotic index was high for 10, in a range from 8 to 65 mitoses per 10 high power field (median, 17.5). Lymphovascular invasion was observed in 51 (82.3%) of 62 patients.

Early Federation of Gynecology and Obstetrics (FIGO) stage disease was noted in 56 patients (IB1, 32; IB2, 10; IIA2, 1; IIB, 13), while advanced FIGO stage disease was noted in 6 (IIIA, 1; IVB, 5). Of the 5 patients with stage IVB, 2 had liver metastasis, 1 had lung metastasis, 1 had liver and para-aortic nodal metastasis, and 1 had metastasis in the pancreas and breast.

### MRI findings

**Figure 1:**
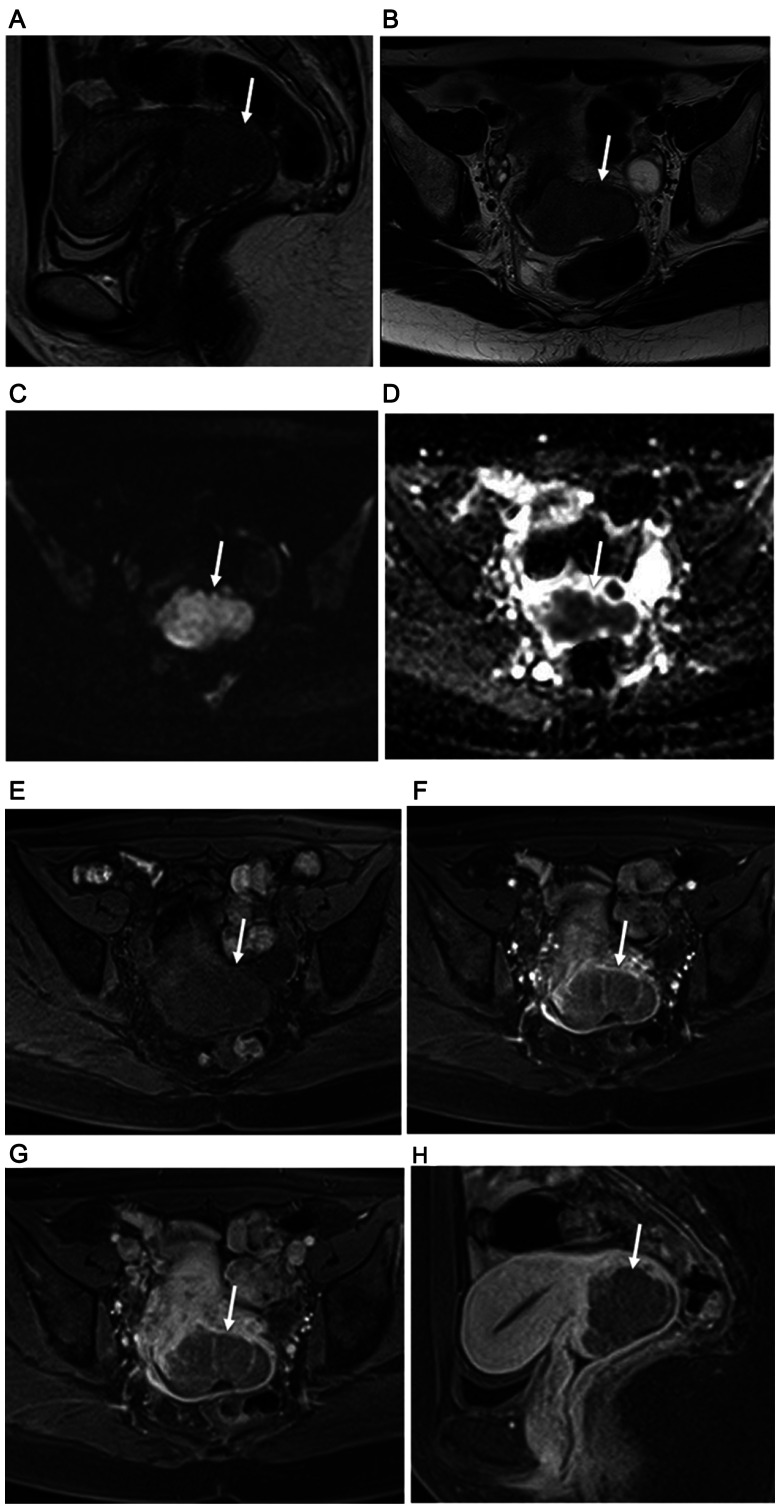
34-year-old woman with FIGO stage IB2 uterine cervical large cell neuroendocrine carcinoma. (**A**) Sagittal and (**B**) axial T2-weighted imagings show homogeneous slightly hyperintense signal from large lobulated mass in uterine cervix (arrow). Subsequent surgery confirmed MRI staging of no invasion of vagina and parametrium. (**C**) Cervical mass with remarkably high signal intensity throughout tumor shown by axial DWI (arrow). (**D**) Obvious diffusion restriction throughout cervical tumor shown by axial ADC mapping (arrow). Results of dynamic contrast-enhanced axial fat-suppressed T1-weighted imaging found at (**E**) pre-contrast stage, and (**F**) 30 and (**G**) 120 seconds after administration of Gd-DTPA show a weak but gradual enhancement pattern (arrows). (**H**) Weak tumor enhancement (arrow) shown by sagittal late-phase enhanced fat-suppressed T1-weighted imaging (240 seconds after Gd-DTPA administration). Recurrence was noted 10.3 months after surgery and death at 20.5 months.

**Figure 2:**
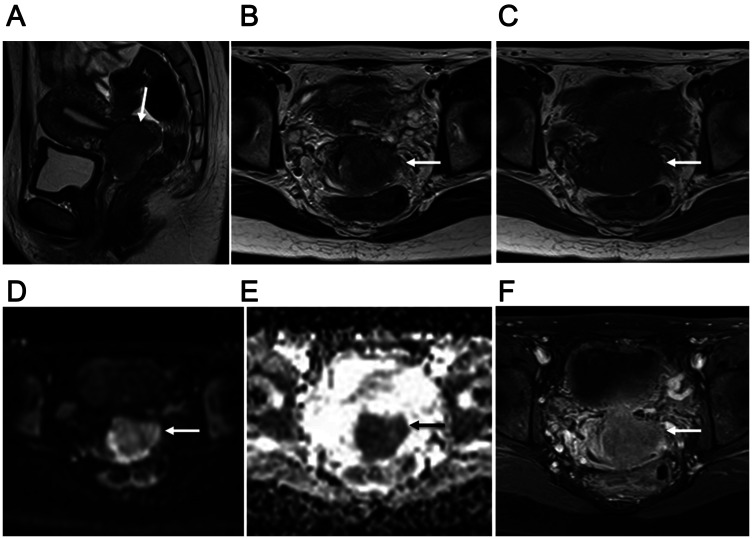
34-year-old woman with FIGO stage IIB2 uterine cervical small cell neuroendocrine carcinoma. (**A**) Lobulated mass with homogeneous and slightly hyperintense signal in the uterine cervix (arrow) shown by sagittal T2-weighted imaging. (**B**) Ill-defined mass with invasion of left parametrium (arrow) in axial T2-weighted image. Subsequent surgery findings confirmed that MRI staging of parametrium invasion was correct. (**C**) Iso-intense mass invading left parametrium (arrow) shown by axial T1-weighted imaging. (**D**) Cervical mass with remarkably high signal intensity (arrow) shown by axial DWI. (**E**) Obvious diffusion restriction throughout tumor (arrow) shown by axial ADC map. (**F**) Weak tumor enhancement (arrow) shown by axial late-phase enhanced fat-suppressed T1-weighted imaging (240 seconds after Gd-DTPA administration). Recurrence was noted 10.8 months after surgery and death at 37.2 months.

**Figure 3:**
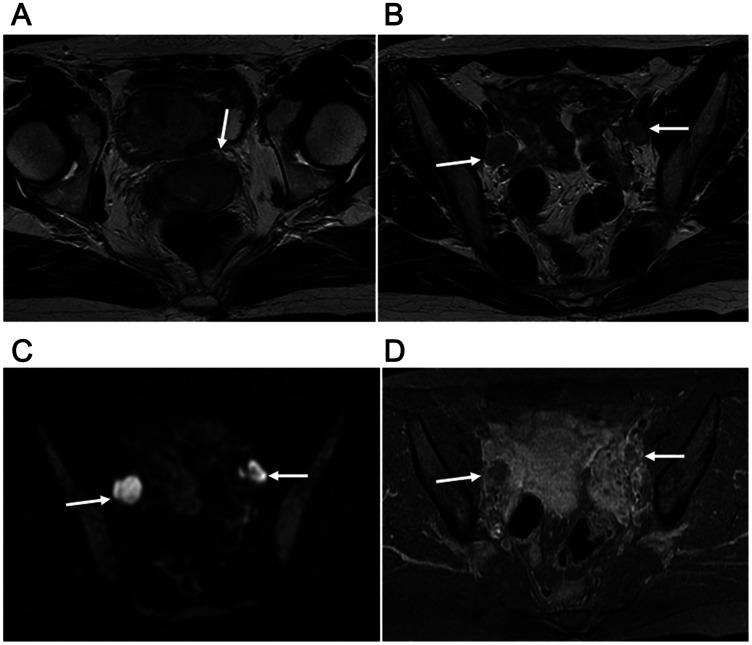
30-year-old woman with T2bN1M0 (stage IIIB) and FIGO stage IIB2 uterine cervical small cell carcinoma mixed with squamous cell carcinoma. (**A**) Ill-defined mass in uterine cervix (arrow) with homogeneous slightly hyperintense signal shown by axial T2-weighted imaging. (**B**) Axial T2-weighted imaging showing 2 swollen lymph nodes in the bilateral obturator areas (arrows), both greater than 1 cm in the short diameter,, suggested to be nodal metastasis. (**C**) Two swollen lymph nodes with high signal intensity (arrows) shown by axial DWI. (**D**) Axial late-phase enhanced fat-suppressed T1-weighted imaging (240 seconds after Gd-DTPA) revealed weak enhancement of the 2 nodes (arrows). Surgery was performed after 2 courses of neoadjuvant chemotherapy, with pathological results showing nodal metastasis. No recurrence was seen at 113.8 months after initial neoadjuvant chemotherapy.

MRI findings are summarized in Table 2. The largest tumor diameter was 36.3 ± 22.4 mm (range, 7–108 mm), with 19 (30.6%) patients showing a largest diameter greater than 4 cm and 43 (69.4%) that at 4 cm or less. Representative cases are presented in Figures 1, 2, and 3.

**Table 2:** MRI findings of cervical neuroendocrine carcinoma

Character	*N*	%
Margin		
Well-defined	55	88.7
Ill-defined	7	11.3
Shape		
Round/Ovoid	51	82.2
Lobulated/Irregular	11	17.7
Internal appearance		
Homogeneous	50	80.6
Heterogeneous	12	19.4
T1-weighted image		
Slightly low	10	16.1
Iso	48	77.4
Slightly high	4	6.5
T2-weighted image		
Slightly high	50	80.6
High	12	19.4
Diffusion weighted imaging		
Intense high	59	100
Apparent diffusion coefficient map		
Intense low	59	100
Dynamic contrast enhancement pattern		
Gradual pattern	30	88.2
Washout/plateau pattern	4	11.8
The enhancement degree on the late phase		
Mild	37	66.1
Moderate	18	32.1
Strong	1	1.8

*N*: number.

### MRI and pathological staging

MRI diagnostic accuracy for tumor extension in a neighboring organ in the 50 patients without NAC is summarized in Table 3. Parametrial invasion was noted in 10 (20.0%) of 50 and correctly detected in 8 (80.0%) patients by MRI (Figure 2). Vaginal invasion was noted in 3 (6.0%) of 50 patients and correctly detected in 2 (66.7%) by MRINo invasion of the pelvic wall, urinary bladder, or rectum mucosa was observed in any of the present patients.

**Table 3:** MRI staging of cervical neuroendocrine carcinoma in 50 patients without NAC on a per patient basis

	Sensitivity	Specificity	PPV	NPV	Accuracy
Invasion of parametrium	80.0% (8/10)	95.0% (38/40)	80.0% (8/10)	95.0% (38/40)	92.0% (46/50)
95% CI	55.2–100	88.2–100	55.2–100	88.2–100	83.6–100
Invasion of vagina	66.7% (2/3)	95.7% (45/47)	50.0% (2/4)	97.8% (45/46)	94.0% (47/50)
95% CI	13.3–100	90.0–100	1.0–99.0	93.6–100	87.4–100
^*^Metastatic pelvic lymph node	38.5% (5/13)	100% (35/35)	100% (5/5)	81.4% (35/43)	83.3% (40/48)
95% CI	12.0–64.9	100	100	69.8–93.0	72.8–93.9

PPV: positive predictive value, NPV: negative predictive value, CI: Confidence interval, ^*^Because 2 patients did not receive pelvic lymphadenectomy, not 50 but 48 patients were analyzed.

According to the most recently presented AJCC cancer staging [8], T stage in 50 patients without neoadjuvant chemotherapy (NAC) was classified as pT1b1 in 32, pT1b2 in 6, pT2a2 in 1, and pT2b in 11. Agreement was noted in 44 patients, with the overall accuracy of MRI local staging 88.0%. Details regarding agreement between MRI T staging and pathological staging are shown in Table 4.

**Table 4:** Correlation between MRI T staging and pathological staging in 50 patients without NAC

	Pathological staging					
MRI staging	T1b1	T1b2	T2a1	T2a2	T2b	All
T1b1	30	0	0	0	1	31
T1b2	0	5	0	1	0	6
T2a1	1	0	0	0	1	2
T2a2	0	0	0	0	0	0
T2b	1	1	0	0	9	11
All	32	6	0	1	11	50

Among the 48 patients who underwent a pelvic lymphadenectomy procedure without NAC, a total of 30 pelvic pathologically positive lymph node (LN) areas were found in 13 patients (27.1%), which consisted of the parametrial (*n* = 2), internal iliac (*n* = 8), external iliac (*n* = 7), common iliac (*n* = 3), and obturator fossa (*n* = 10) areas. In LN area-by-area analyses, the sensitivity, specificity, positive predictive value (PPV), negative predictive value (NPV), and accuracy for detecting metastatic pelvic LN areas were 33.3% (10/30), 100% (450/450), 100%, (10/10) 95.7% (450/470), and 95.8% (460/480), respectively. As for patient-based sensitivity, specificity, PPV, NPV, and accuracy for detecting metastatic pelvic LN, those values were 38.5% (5/13), 100% (35/35), 100% (5/5) 81.4% (35/43), and 83.3% (40/48), respectively (Table 3) (Figure 3).

### Survival analysis

Following surgery, 38 patients (31 without, 7 with NAC) received adjuvant chemotherapy and 13 (11 without, 2 with NAC) received chemoradiation therapy, while 11 patients (8 without, 3 with NAC) underwent no therapy. During a follow-up period of 45.6 months (4.3–151.0 months), 28 (45.2%) of 62 patients experienced recurrence or progression, including 15 (35.7%) of 42 classified as FIGO I, 7 (50.0%) of 14 as FIGO II, 1 (100%) of 1 as FIGO III, and 5 (100%) of 5 as FIGO IV. The sites of recurrence in those 28 patients were lung (*n* = 4), liver (*n* = 3), lung and liver (*n* = 3), lung, liver, and bone (*n* = 3), local (*n* = 2), LN (*n* = 2), local and LN (*n* = 2), liver and bone (*n* = 2), pancreas (*n* = 1), lung and bone (*n* = 1), LN and bone (*n* = 1), peritoneum and bone (*n* = 1), local, liver, and bone (*n* = 1), LN, lung, and bone (*n* = 1), and LN, lung, and liver (*n* = 1).

**Figure 4:**
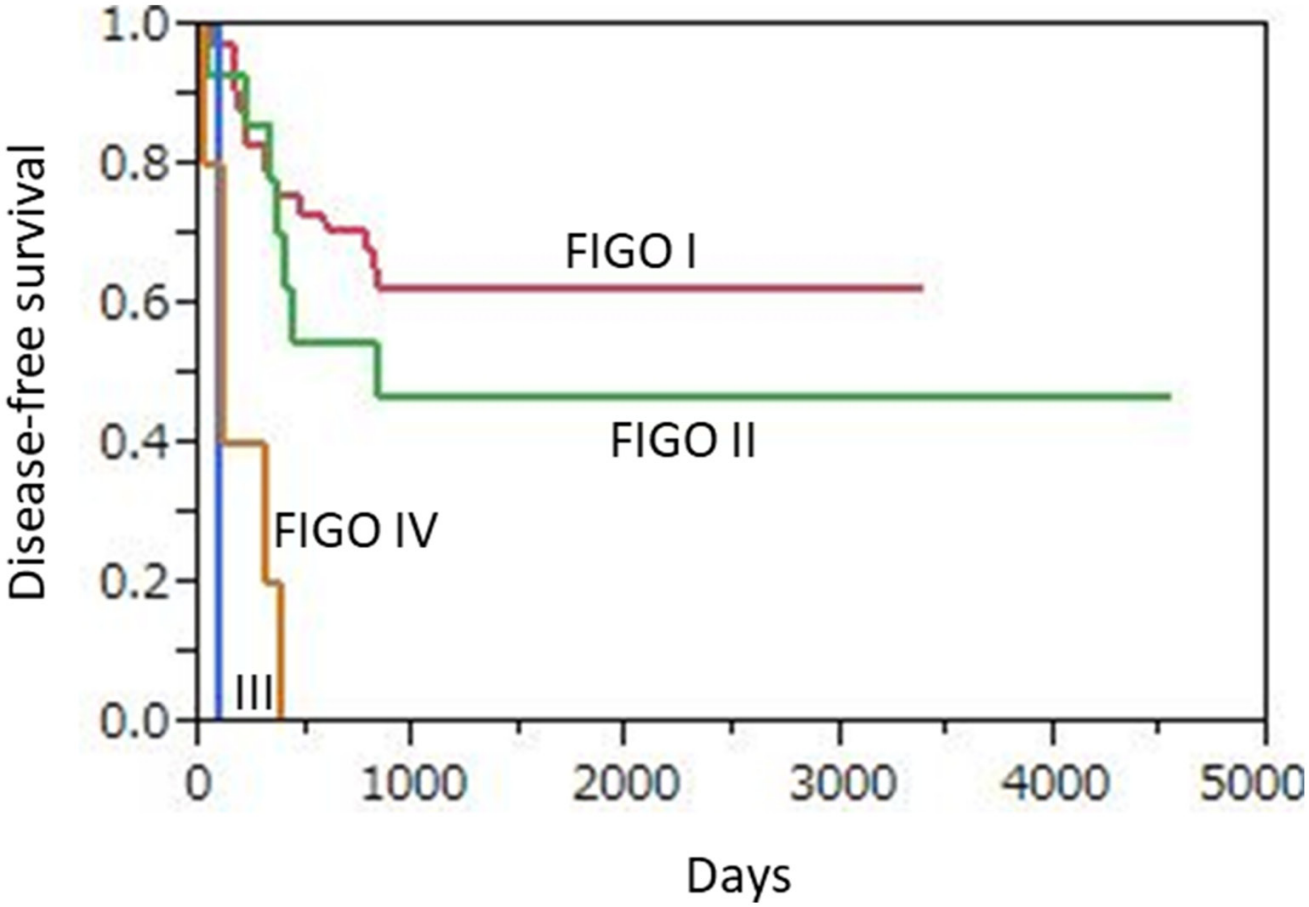
Kaplan-Meier disease-free survival curves for 62 uterine cervical neuroendocrine carcinoma patients according to FIGO stage. Advanced FIGO staging disease (III, IV) patients (*n* = 6) showed significantly shorter PFS as compared to those (*n* = 56) with early FIGO staging disease (I, II) (*p* < 0.0001).

The median period from surgery to recurrence or progression for those 28 patients was 9.85 months (range 1.1 to 27.8 months). As for 3-year progression-free survival (PFS), those rates were 64.3% (27/42) for FIGO I, 50.0% (7/14) for FIGO II, 0% (0/1) for FIGO III, and 0% (0/5) for FIGO IV, while that was 54.8% (34/62) for all patients (Figure 4). Of those, 6 patients with advanced FIGO stage disease (III, IV) showed a shorter PFS than the 56 with early FIGO stage disease (I, II) and the difference was significant (*p* < 0.0001).

**Figure 5:**
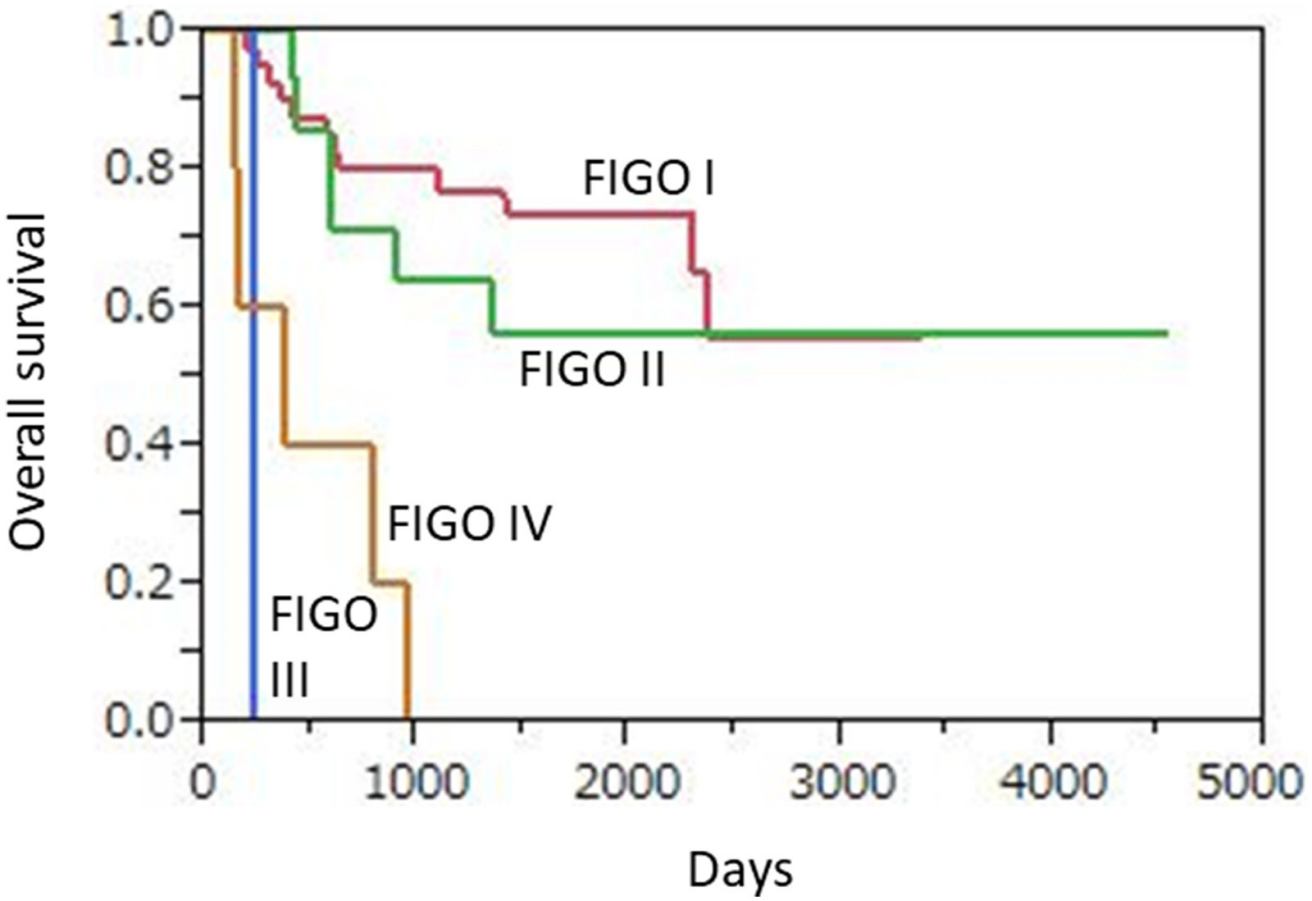
Kaplan-Meier overall survival curves for 62 uterine cervical neuroendocrine carcinoma patients according to FIGO stage. Advanced FIGO staging disease (III, IV) patients (*n* = 6) showed significantly worse OS as compare to those (*n* = 56) with early FIGO staging disease (I, II) (*p* < 0.0001).

During the follow-up period, 24 (38.7%) died of disease, including 12 (28.6%) of those classified as FIGO I, 6 (42.9%) as FIGO II, 1 (100%) as FIGO III, and 5 (100%) as FIGO IV. Median OS was 43.3 months for all patients, and 47.1, 46.6, 7.8, and 12.3 months when divided into FIGO I, II, III, and IV cases, respectively. The 3-year overall survival (OS) rates based on FIGO classification were 80.9% (34/42), 64.3% (9/14), 0% (0/1), and 0% (0/5), respectively, and 69.4% (43/62) for all patients (Figure 5). The 6 patients with advanced FIGO stage (III, IV) showed significantly worse OS than the 56 patients with early FIGO stage (I, II) disease (*p* < 0.0001).

## DISCUSSION AND CONCLUSIONS

This analysis of 62 patients with uterine cervical NEC proven by histological and surgery results revealed important findings related to MRI and patient prognosis. We found that likely suggestive features of uterine cervical NEC are a homogeneous lesion texture with an obvious restricted diffusion throughout the tumor, and consider that MRI is reliable for T staging of affected patients. Furthermore, it was confirmed that uterine cervical NEC is related to poor prognosis, especially in an advanced stage.

Two previous reports have presented MRI findings of patients with uterine cervical SCNEC [6, 7]. The present results were similar to those, as follows. First, advanced disease of the cervical NEC exhibited an ill-defined margin, irregular shape, infiltration into adjacent structures, and extensive lymphadenopathy in MRI results. These tumors also showed a significant tendency for homogeneous signal intensity with T2WI and homogeneous enhancement. Additionally, DWI showed abnormal high signal intensity with low ADC values throughout the tumor, reflecting a high level of cellularity of malignant cells. Duan et al. [7] reported that these MRI findings are nonspecific for indicating tumor malignancy, and that differentiation between squamous cell carcinoma and adenocarcinoma using MRI results is not easy in cases of cervical NEC. On the other hand, they also noted that lesion homogeneity and low ADC values shown by MRI are likely features suggestive of cervical NEC and the accuracy of MRI local T staging was very high (85.7%), very similar to the value (88.0%) noted in our study.

An NEC of the uterine cervix is aggressive and shows a highly malignant clinical behavior [3, 4]. Wang et al. [9] evaluated survival of 31 affected patients and reported a mean survival time of 32.2 months, with 2- and 5-year OS rates of 54.8% and 31.5%, respectively. Zivanovic et al. [10] evaluated survival of 17 patients with uterine cervical SCNEC and found a median OS of 14.6 months, along with 3-year relapse-free survival (RFS) and overall survival (OS) rates of 22% and 30%, respectively. In that study, the extent of disease was the only significant prognostic factor, and median OS was 31.2 months for patients with early stage (IA- IB2) and 6.4 months for those with advanced stage (IIB-IV) disease (*p* = 0.034). The same poor prognosis in advanced stage cases was identified in our series, as the 3-year OS rate for FIGO I and II was 76.8%, while that was 0% for FIGO III and IV cases. In the study by Wang et al. [9], the age of 31 patients with uterine cervical NEC was 42 ± 11.3 years, while that of the 25 reported by Duan et al. [7] was 46.3 ± 10.3 years (range 26–69 years). The mean age in the present cohort (43.5 years) was similar to those previous reports.

Although a clear treatment protocol for NEC of the uterine cervix has not been presented, it is recommended that patients be given multimodality therapy, including surgery, chemotherapy, and radiotherapy [4], with surgery generally thought to have the greatest impact. Zivanovic et al. [10] demonstrated that patients who received platinum-based chemotherapy had a 3-year RFS of 83% and OS as well of 83%, whereas those for patients who did not receive chemotherapy were 0% and 20%, respectively.

The present study results are limited by the retrospective design and variations in the MRI scan protocols used among the participating centers over the 13-year study period due to the rarity of this entity. The pathological diagnosis of 62 cervical NECs was made by each pathologist at each institution, not by one pathologist (central diagnosis).

In conclusion, NEC of the uterine cervix is rare and affected patients have a poor prognosis, especially advanced stage cases. A definitive diagnosis based on preoperative MRI results seems to be difficult. Nevertheless, the present as well as other previous findings indicate that a homogeneous lesion texture with an obvious restricted diffusion throughout the tumor are suggestive of uterine cervical NEC. Furthermore, we found pelvic MRI to provide reliable imaging findings for T staging in these patients.

## MATERIALS AND METHODS

### Patient recruitment

This retrospective multicenter study of MRI findings of uterine cervical NEC included 24 institutions, and the appropriate review board at each gave approval to the protocol and waived the requirement for patient-informed consent. Sixty-two patients (average age at diagnosis 43.5 years, range 26–82 years) with uterine cervical NEC underwent pre-treatment pelvic MRI examinations between May 2006 and May 2018 at one of the participating institutions. For whole-body staging, 58 underwent chest/abdomen/pelvis computed tomography (CT) and 4 ^18^F-fluorodeoxyglucose-positron emission tomography/computed tomography (FDG-PET/CT). Twelve (19.4%) of the 62 patients received neoadjuvant chemotherapy in 2 (*n* = 8), 3 (*n* = 3), or 4 (*n* = 1) courses. Of 50 patients without NAC, 2 underwent abdominal hysterectomy and bilateral salpingo-oophorectomy, 41 abdominal hysterectomy, bilateral salpingo-oophorectomy, and pelvic lymphadenectomy, and 7 abdominal hysterectomy, bilateral salpingo-oophorectomy, pelvic lymphadenectomy, and para-aortic lymphadenectomy procedures. As for the 12 patients with NAC, 1 underwent abdominal hysterectomy and bilateral salpingo-oophorectomy, 7 abdominal hysterectomy, bilateral salpingo-oophorectomy, and pelvic lymphadenectomy, and 4 abdominal hysterectomy, bilateral salpingo-oophorectomy, pelvic lymphadenectomy and para-aortic lymphadenectomy procedures. Conventional haematoxylin eosin and immunochemistry staining for various markers was applied to resected tumors and biopsy samples, including synaptophysin, chromogranin A, CD56, NSE, p53, SSRT2a, and Ki-67. Each tumor was staged according to the 2008 International FIGO staging system for carcinomas of the uterine cervix [11].

### Pelvic MRI

Pelvic MRI examinations were performed using equipment available at the participating institutions during the course of this study, including a 1.5-T system (1.5T Gyroscan Intera NT, or Intera Achieva 1.5T nova dual; Philips Medical Systems, Best, The Netherlands, Optima MR450w, SIGNA HDxt 1.5T, or SIGNA EXCITE; GE Healthcare, Waukesha, WI, USA, MAGNETON Vision, MAGNETOM Avanto, or MAGNETOM ESSENZA; Siemens Medical Solutions, Erlangen, Germany, NT MRT200SP5; Canon Medical System, Ohtawara, Japan) in 36 patients or a 3-T system (Achieva 3.0T TX, Intera Achieva Quasar dual, or Ingenia CX; Philips Medical Systems, Best, The Netherlands, DISCOVERY MR750w or SIGNA EXCITE 3.0T-HD; GE Healthcare, Waukesha, WI, USA, MAGNETON Prisma, MAGNETON Skyra or MAGNETON Trio; Siemens Medical Solutions, Erlangen, Germany, Vantage Titan 3T; Cannon Medical System, Ohtawara, Japan) in 26 patients. For those examinations, a body coil was used for excitation and a pelvic phased-array coil for signal reception. Butyl scopolamine (Buscopan, Boehringer Ingelheim) was given intramuscularly to all patients immediately before the examination to reduce artifacts from bowel peristalsis, unless contraindicated. The MRI parameters varied depending on the institution. Unenhanced axial and sagittal fast-spin-echo T2WI was performed with a 4–5 mm slice thickness, and unenhanced T1WI results were acquired in the axial and sagittal planes with a spin-echo, and 4- to 5-mm slice thickness, In 59 patients, axial DWI was performed in 3 orthogonal directions using spin-echo-type single-shot echo planar imaging with a 4- to 5-mm slice thickness. Fifty-six patients underwent contrast-enhanced scanning. In 34 patients, following administration of 0.1 mmol/kg gadolinium diethylenetriaminepentaacetic acid (Gd-DTPA) at a rate of 2.0–3.0 ml/second, followed by a saline flush (15 ml at 2.0–3.0 ml/second), multiphase dynamic images with fast-gradient-echo, fat-suppressed T1-weighted, axial, or sagittal sequences (2- to 3-mm slice thickness) were obtained. Delayed (4 to 6 minutes after Gd-DTPA administration) T1-weighted fat-suppressed axial and sagittal sequences were obtained sequentially, with parameters similar to those used before injection of Gd-DTPA. In the other 22 patients, following a single injection of Gd-DTPA (0.1 mmol/kg body weight), T1-weighted fat-suppressed axial and sagittal sequences in the late phase were obtained sequentially, using parameters similar to those used prior to injection of Gd-DTPA.

### Imaging analysis

Pelvic MRI results were reviewed by 2 experienced radiologists with experience in gynecological MRI (K. K., 18 years; Y. K., 10 years) and blinded to patient information. Their decisions were made in a consensus manner. For expert review, MR images were displayed with a Digital Imaging and Communications in Medicine viewer (Osirix; Pixmeo, Geneva, Switzerland), and assessments of tumor location, size, margin (well-defined or ill-defined), shape (round/ovoid or lobular/irregular), signal intensity (compared with uterine myometrium), lesion texture (homogeneous or heterogeneous), contrast enhancement patterns, and degree of enhancement were made. Tumor size was measured as the greatest diameter in either the transverse or sagittal plane. Dynamic contrast enhancement patterns were categorized as washout/plateau or gradual. Time-intensity curves were categorized into progressive, plateau, washout, and indeterminate types. Progressive enhancement was defined as a gradual increase in signal throughout all phases of enhancement. Plateau enhancement was defined as an initial increase in signal followed by a plateau in which the MRI signal units remained unchanged within a range of a 5% difference. A washout curve was defined as a steep initial increase and peak in signal followed by a decrease of at least 5%–10% [12]. The degree of tumor enhancement in the late phase was compared with normal myometrium enhancement, then classified as mild, moderate, or strong.

The experienced readers also evaluated tumor extension into the parametrium, vagina, pelvic wall, and urinary bladder or rectum mucosa as well as pelvic lymph nodes (LNs), using methods detailed in previous reports [13, 14]. Lymphadenopathy location was defined as right or left parametrial, internal iliac, external iliac, common iliac, or obturator area. Lymphadenopathy was considered to be present based on size criteria (> 1 cm in short diameter) and/or signal intensity (with central necrosis).

### Statistical analysis

Values are presented as the mean ± standard deviation (SD) and number (%). PFS was analyzed using Kaplan-Meier plots and a log-rank test, and defined as time from initial treatment to tumor recurrence or progression, determined by histologic or follow-up findings, including imaging results. OS was defined as time from initial treatment to date of death from the disease. Statistical analysis was performed using SAS, version 9.3 (SAS Institute Inc.). *P* values < 0.05 were considered to indicate significance.

## Abbreviations

NEC: neuroendocrine carcinoma
NAC: neoadjuvant chemotherapy
WHO: World Health Organization
SCNEC: small cell neuroendocrine carcinoma
LCNEC: large cell neuroendocrine carcinoma
MRI: magnetic resonance imaging
NEC: neuron-specific enolase
FIGO: Federation of Gynecology and Obstetrics
T1WI: T1-weighted imaging
T2WI: T2-weighted imaging
ADC: apparent diffusion coefficient
DWI: diffusion weighted imaging
LN: lymph node
PPV: positive predictive value
NPV: negative predictive value
PFS: progression-free survival
OS: overall survival
RFS: relapse-free survival
SD: standard deviation
